# Food insecurity status and perceived subjective well-being by maternal age and pregnancy status among women in Nigeria: a cross-sectional multilevel analysis

**DOI:** 10.1017/S1368980026102535

**Published:** 2026-04-10

**Authors:** Otobo I. Ujah, Olamide Asifat, Teniola Akinosho, Innocent A.O. Ujah, Russell Kirby

**Affiliations:** 1 Department of Obstetrics and Gynaecology, Federal University of Health Sciences, Otukpo, Nigeria; 2 Georgia Southern University, USA; 3 University of New Haven, USA; 4 Federal University of Health Sciences Otukpo, Nigeria; 5 University of South Florida College of Public Health, USA

**Keywords:** Food insecurity, Subjective well-being, Pregnancy, Postpartum, Mental Health

## Abstract

**Objective:**

To examine the association between household food insecurity (HFI) and low subjective well-being (SWB) among pregnant and postpartum women and determine whether these potential associations differed by maternal age and pregnancy status.

**Design:**

We conducted a secondary analysis of nationally representative cross-sectional data from women of reproductive age (15–49 years). HFI was measured using the Food Insecurity Experience Scale and categorised as none/mild, moderate or severe. Weighted multilevel logistic regression models were used to estimate OR and 95 % CI for the association between HFI and low levels of three SWB measures: happiness, life satisfaction and optimism. Analyses were stratified by age and pregnancy status.

**Setting:**

Data were drawn from the 2021 Nigeria Multiple Indicator Cluster Survey, Round 6.

**Participants:**

The analytic sample comprised 12 587 women who were pregnant at the time of the survey or within 24 months postpartum.

**Results:**

HFI was significantly associated with all three measures of SWB, although the magnitude of associations varied by outcome, even after adjusting for individual-, household-and community-level characteristics. Stratified analyses revealed heterogeneity in the associations between HFI and SWB by age and pregnancy status. Overall, HFI was associated with lower levels of happiness, life satisfaction and optimism among pregnant and postpartum women in Nigeria.

**Conclusions:**

Our findings demonstrate a negative association between HFI and SWB among pregnant and postpartum women in Nigeria. These associations were modified by maternal age and pregnancy status, suggesting that strategies to mitigate HFI should account for subgroup differences in order to effectively improve maternal well-being.

Food insecurity (FI) has emerged as an important public health concern globally. According to the FAO of the UN, FI occurs when individuals or households experience limitations in securing access to sufficient, safe and nutritious food, thereby impairing normal growth, development and the ability to maintain an active and healthy life^(1,2)^. In 2023, nearly one-third (28·9 %) of the global population experienced FI^(3)^. In West Africa alone, approximately 270 million individuals were food-insecure, with slightly more than one-third experiencing severe FI^(3)^.

As a multidimensional construct, FI hierarchically encompasses food availability, access (both economic and physical), utilisation and temporal stability^(4–6)^. Food availability reflects the supply-side factors that determine food sufficiency, including the quantity and quality of food produced and distributed within a system^(6)^. Food access, in contrast, represents the demand side of food security and refers to the ability of individuals or households to obtain adequate food – either through their own production or by purchasing it^(5,7)^. Food utilisation involves the biological and social processes through which food is converted into positive health outcomes^(6)^, while temporal stability captures the consistency of these three dimensions over time, recognising that FI can be transitory, cyclical or chronic in nature^(5)^.

Food insecurity has negative implications for nutritional status, leading to inadequate dietary intake, reduced dietary diversity and increased risk of micronutrient deficiencies^(3)^. Studies have shown that FI is associated with a paradoxical vulnerability to overweight and obesity, particularly among women in high-income countries^(8,9)^. Also, in low-and middle-income countries, FI has been linked to anaemia and inadequate dietary diversity among women of reproductive age^(10,11)^.

Beyond its nutritional impacts, there is growing recognition of the broader, non-nutritional consequences of FI^(12,13)^. One of these is its effect on subjective well-being (SWB) – a key dimension of quality of life that captures individuals’ emotional experiences (affective well-being), life satisfaction (cognitive well-being) and optimism about the future (vitality and hope)^(14,15)^. While health and well-being are conceptually distinct, they are intricately interrelated and mutually reinforcing^(16,17)^. From a public health perspective, measures of SWB have been increasingly used as proxies for population-level mental health^(18)^. Emerging evidence indicates that FI is associated with lower life satisfaction, reduced quality of life and overall declines in SWB among adults and older populations^(19,20)^.

Women are disproportionately affected by FI, particularly during pregnancy and the postpartum period^(21,22)^. A global analysis of data from 142 countries shows that women living in food-insecure households have higher odds of reporting low life satisfaction^(23)^. In addition, higher prenatal eudaimonic well-being has been linked to longer gestational age and higher birth weight^(24)^, while poor maternal well-being has been associated with maladaptive health and caregiving behaviours during pregnancy and the postpartum period, including unhealthy dietary practices, reduced self-care and impaired mother–infant bonding^(25,26)^.

Despite increasing recognition that FI contributes to disparities in SWB, there remains a lack of empirical evidence, particularly in low-and middle-income countries (LMICs), delineating how FI interacts with maternal age and pregnancy status to shape SWB during pregnancy and postpartum. To overcome these research gaps, we sought to examine the relationship between FI and measures of SWB, stratified by maternal age and pregnancy status among a nationally representative sample of women in Nigeria. We hypothesised that FI would be associated with higher odds of reporting low levels of SWB and these associations would vary by age and pregnancy status.

## Methods

### Study design, data source and study population

We performed a secondary analysis of data drawn from Round 6 (2021) of the UNICEF-supported Multiple Indicator Cluster Surveys (MICS) in Nigeria. The MICS is a household survey developed to collect data on health and social indicators from representative samples of children, women and men in low-and middle-income countries. The survey employed a multistage stratified cluster sampling approach to sample respondents for the survey. Details of the survey sampling design and data collection techniques are described elsewhere^(27)^. For this study, we merged the household files, which contained the variables related to FI, with the women’s files which contained variables related to SWB. We used the national women’s sample survey weights to report the survey results to account for the complex sampling design.

### Sample selection

Our analytic sample comprised pregnant and postpartum women (within 24 months of delivery) aged 15–49 years (unweighted *n* 12 899). To ascertain women’s pregnancy status, two questions were used to ascertain the eligibility: ‘*Are you pregnant now?*’ (Yes, No) and ‘*Was there a live birth in the last 2 years?*’ (Yes, No). Further, we excluded observations with missing observations on the relevant variables as follows: happiness (*n* 4), life satisfaction (*n* 15), optimism (*n* 58), FI (*n* 99), marital or cohabiting status (*n* 7) and religion (*n* 2). The final analytic sample comprised 12 587 eligible participants nested in 12 718 clusters.

### Measures

Household food insecurity (HFI) was the main explanatory variable. The MICS assessed HFI using the eight-item Food Insecurity Experience Scale (FIES) developed by the UN FAO^(28)^. Participants were asked a series of questions regarding their HFI status in the past 12 months. The response options for each FIES question included ‘*Yes*’, ‘No’ or ‘*Don’t know*’. Composite household HFI scores were computed by summing up the affirmative responses, resulting in a score range of 0–8. We operationalised HFI based on FIES score as none/mild (0–3), moderate (4–6) and severe HFI (7–8)^(29,30)^.

We assessed three measures of SWB outcomes – happiness, life satisfaction and optimism. To assess happiness, participants were asked the following question: ‘*I would like to ask you some simple questions on happiness and satisfaction. First, taking all things together, would you say you are very happy, somewhat happy, neither happy nor unhappy, somewhat unhappy or very unhappy?*’. The item was answered on a five-point Likert scale ranging from 1 (very unhappy) to 5 (very happy). As in a previous study^(31)^, we modelled happiness as a dichotomous variable with the responses categorised as high (responses with very happy or somewhat happy) and low (responses with somewhat unhappy, neither happy nor unhappy or very unhappy).

Life satisfaction was assessed in the MICS using Cantril’s Self-Anchoring Ladder, a widely used and validated instrument developed by Hadley Cantril in 1965 to assess individuals’ overall life satisfaction. During the survey, participants were asked ‘*Now, look at this ladder with steps numbered from 0 at the bottom to 10 at the top. Suppose we say that the top of the ladder represents the best possible life for you and the bottom of the ladder represents the worst possible life for you. On which step of the ladder do you feel you stand at this time?*’. Higher values indicated better life satisfaction. Life satisfaction in this study was modelled as a dichotomous variable. Life satisfaction scores of 6–10 were defined as high life satisfaction and 0–5 as low^(32,33)^.

To measure optimism, participants were asked, ‘*In one year from now, do you expect that your life will be better, will be more or less the same, or will be worse, overall?*’ Responses were dichotomised as high (will be better) *v.* low (i.e. will be more or less the same, or will be worse, overall)^(32)^.

To account for potential confounding, we adjusted for selected covariates identified following a comprehensive literature review, theoretical significance and biological plausibility^(21,34,35)^. The individual/household-level characteristics considered in the analysis included age, religion, marital status, number of birth events, pregnancy status, pregnancy intention, health insurance, household size and household wealth index. The community-level characteristics considered were place of residence and region of residence.

We also performed subgroup analyses by stratifying the sample into different subpopulations based on age groups (15–24 years, 24–34 years and 35–49 years) and by pregnancy status (pregnant and postpartum).

### Statistical analysis

We performed statistical analyses using SAS software (version 9.4), while figures were prepared using R (version 4.3.2). We performed a complete case analysis as approximately 1·4 % of data was missing for all variables. To account for the complex sampling design, including cluster and stratification information, we applied the survey weights included in the MICS data to compute weighted means and standard errors for continuous variables and frequencies and weighted percentages for categorical variables, respectively. Rao-Scott χ^2^ analyses were performed to test for between-group differences in the distribution of background characteristics overall and by measures of SWB.

We fitted separate weighted multilevel logistic regression models (with random intercepts) to explore the relationship between FI and SWB, controlling for individual-, household-and contextual-level characteristics. We employed this approach as the MICS data were hierarchically structured with women *i* (Level 1) nested in communities *j* (Level 2) and given the dichotomous nature of the outcome variables. To test our hypotheses, we defined four 2-level random intercept-only models *a priori* as well as specifying a null (unconditional) model. Thereafter, we built complex conditional models by sequentially adding the main explanatory variable of interest and Level 1 and Level 2 predictors. In model I (crude model), we first estimated the main effect of the FI on each self-reported measure of SWB, model II included model I and adjusted for individual-/household-level variables only and model III included model I adjusted for community-level variables. In the final model (model IV), we included model I and adjusted for both individual-/household-level and community-level variables. None/mild FI status was the reference group to make comparisons to moderate and severe FI status. Additionally, we conducted separate analyses stratified by maternal age and by pregnancy status.

The fixed-effects estimates are presented as crude and adjusted OR along with their corresponding 95 % CI. All tests were two-tailed, and a *p* value of 0·05 was used to determine statistical significance. To examine contextual effects, we estimated the intraclass correlation coefficient (ICC) and median OR (MOR).

The intraclass correlation coefficient (ICC) quantifies the variability in low SWB attributed to the community level. The within-community variance in logistic regression models is represented by the variance of the standard logistic distribution. By using the logistic distribution variance of approximately 3·29 (or π^2^/3), the ICC is computed as shown below:

ICC = 100 × (τ_00_/(τ_00_ + 3·29)), where τ_00_ is the between-community variance.

The MOR, on the other hand, quantifies the variability between communities by comparing two individuals randomly selected from different communities. In the context of SWB, the MOR can be interpreted as the relative increase in the odds of reporting lower levels of SWB associated with moving from a community with lower levels of SWB to one with higher levels of SWB when two individuals with the same characteristic values are randomly selected from two different communities from our sample^(36,37)^. It is calculated based on the following equation:

MOR = exp(√2 × τ_00_ × 0·6745) = exp(0·95√τ_00_), where 0·6745 is the 75th percentile of the cumulative distribution function of the normal distribution^(38)^. Values equal to 1 suggest no heterogeneity, while values greater than 1 suggest larger heterogeneity^(37)^. Different models were compared using measures of goodness of fit, including the Akaike information criterion and the Bayesian information criterion. Models with smaller Akaike information criterion and Bayesian information criterion values indicate better fitting models.

## Results

### Characteristics of the study population

The sample comprised 3508 pregnant (26·9 %) and 9210 postpartum (73·1 %) women distributed across 1727 communities. Approximately three-quarters of the sample were aged 34 years or younger. Table 1 shows the descriptive statistics for the analytic sample. Overall, approximately one in ten (8·3 %) of respondents reported low optimism. About one in five (20·4 %) of respondents reported low happiness, and approximately 39·3 % reported low life satisfaction. Most participants were either married/living with a partner or cohabitating (95·4 %), and approximately two-thirds were affiliated with religions other than Christianity. Additionally, most lived in poor households (47·6 %), and 71 % were living with at least five members in the household. Most participants (66·4 %) resided in urban areas, and about 35·4 % were residing in the southern region of Nigeria.

**Table 1.** Characteristics of the study population (overall and stratified by SWB measures), Multiple Indicator Cluster Survey (MICS), Nigeria, 2021

	Measures of SWB																
			Happiness					Life satisfaction					Optimism				
	Overall		Low		High			Low		High			Low		High		
Variables	*n*	%	*n*	%	*n*	%	*P* value^b^	*n*	%	*n*	%	*P* value^b^	*n*	%	*n*	%	*P* value^b^
Total	12 718	100	2703	20·4	10 015	79·6	< 0·0001	5085	39·3	7633	60·7	< 0·0001	1226	8·3	11 492	91·7	< 0·0001
Individual and household characteristics																	
Age group, years (range: 15–49)																	
15–24	3650	27·1	736	25·6	2914	27·5	0·11	1427	26·5	2223	27·5	0·29	351	28·1	3299	27·0	0·85
25–34	5961	46·9	1260	46·5	4701	47·1		2357	46·7	3604	47·2		583	46·6	5378	47·0	
35–49	3107	25·9	707	27·9	2400	25·4		1301	26·9	1806	25·3		292	25·3	2815	25·9	
Pregnancy status																	
Pregnant	3508	26·9	727	26·0	2781	27·1	0·69	1381	25·8	2127	27·6	0·08	382	31·5	3126	26·4	0·01
Postpartum	9210	73·1	1976	74·0	7234	72·9		3704	74·2	5506	72·4		844	68·5	8366	73·6	
Marital status																	
Single	613	4·6	260	9·7	353	3·3	< 0·0001	358	7·3	255	2·9	< 0·0001	79	6·8	534	4·4	0·06
Married/living with partner	12 105	95·4	2443	90·3	9662	96·7		4727	92·7	7378	97·2		1147	93·2	10 958	95·6	
Religion																	
Non-Christian	8033	61·7	1744	63·7	6289	61·1	0·14	3294	62·9	4739	60·9	0·19	877	71·3	7156	60·8	
Christian	4685	38·3	959	36·3	3726	38·9		1791	37·1	2894	39·1		349	28·7	4336	39·2	0·0001
Number of household members																	
< 5	3416	29·2	666	26·4	2750	29·9	0·01	1274	26·2	2142	31·2	< 0·0001	267	22·6	3149	29·8	0·0003
≥ 5	9302	70·8	2037	73·6	7265	70·1		3811	73·8	5491	68·8		959	77·4	8343	70·2	
Household wealth index																	
Quintile 1 (lowest)	3629	24·3	806	26·5	2823	23·7	< 0·0001	1506	25·3	2123	23·6	< 0·0001	419	30·4	3210	23·7	< 0·0001
Quintile 2	3257	23·3	730	25·1	2527	22·9		1368	25·5	1889	21·9		327	25·3	2930	23·2	
Quintile 3	2593	18·9	631	21·6	1962	18·2		1089	19·9	1504	18·2		238	20·5	2355	18·8	
Quintile 4	1908	17·0	391	17·8	1517	16·8		747	17·1	1161	16·9		172	16·7	1736	17·0	
Quintile 5 (highest)	1331	16·5	145	9·1	1186	18·4		375	12·1	956	13·1		70	7·3	1261	17·3	
Contextual factors																	
Residence																	
Rural	3315	35·6	635	33·6	2680	36·0	0·24	1296	34·2	2019	36·4	0·19	287	30·6	3028	35·9	0·07
Urban	9403	64·4	2068	66·4	7335	64·0		3789	65·8	5614	63·6		939	69·4	8464	65·0	
Region																	
North Central	2421	14·2	487	14·0	1934	14·2	0·001	875	12·8	1546	15·1	< 0·0001	168	13·4	2253	14·3	< 0·0001
North East	2988	16·2	538	13·7	2450	16·9		1335	18·3	1653	14·9		472	30·6	2516	14·9	
North West	3848	34·2	944	38·7	2904	33·0		1604	35·5	2244	33·3		344	33·3	3504	34·3	
South East	1097	8·3	259	9·8	1097	10·4		447	9·7	650	7·4		155	11·2	942	8·0	
South South	1222	11·1	274	10·6	948	11·3		444	10·6	778	11·5		25	3·8	1197	11·8	
South West	1142	16·0	201	13·1	941	16·7		380	13·1	762	17·8		62	7·6	1080	16·7	

SWB, subjective well-being.

Data are presented as unweighted sample frequencies (*n*) with weighted population proportions (%). Percentage may not sum to 100 due to rounding.

**Figure 1.**
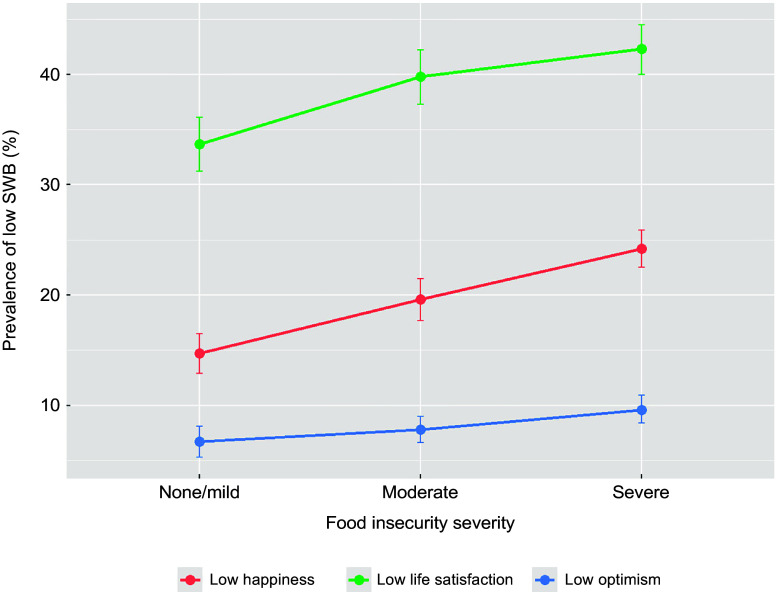
Prevalence of low levels of subjective well-being indicators by food insecurity status among pregnant and postpartum women in Nigeria. Error bars show 95 % CI.

**Figure 2.**
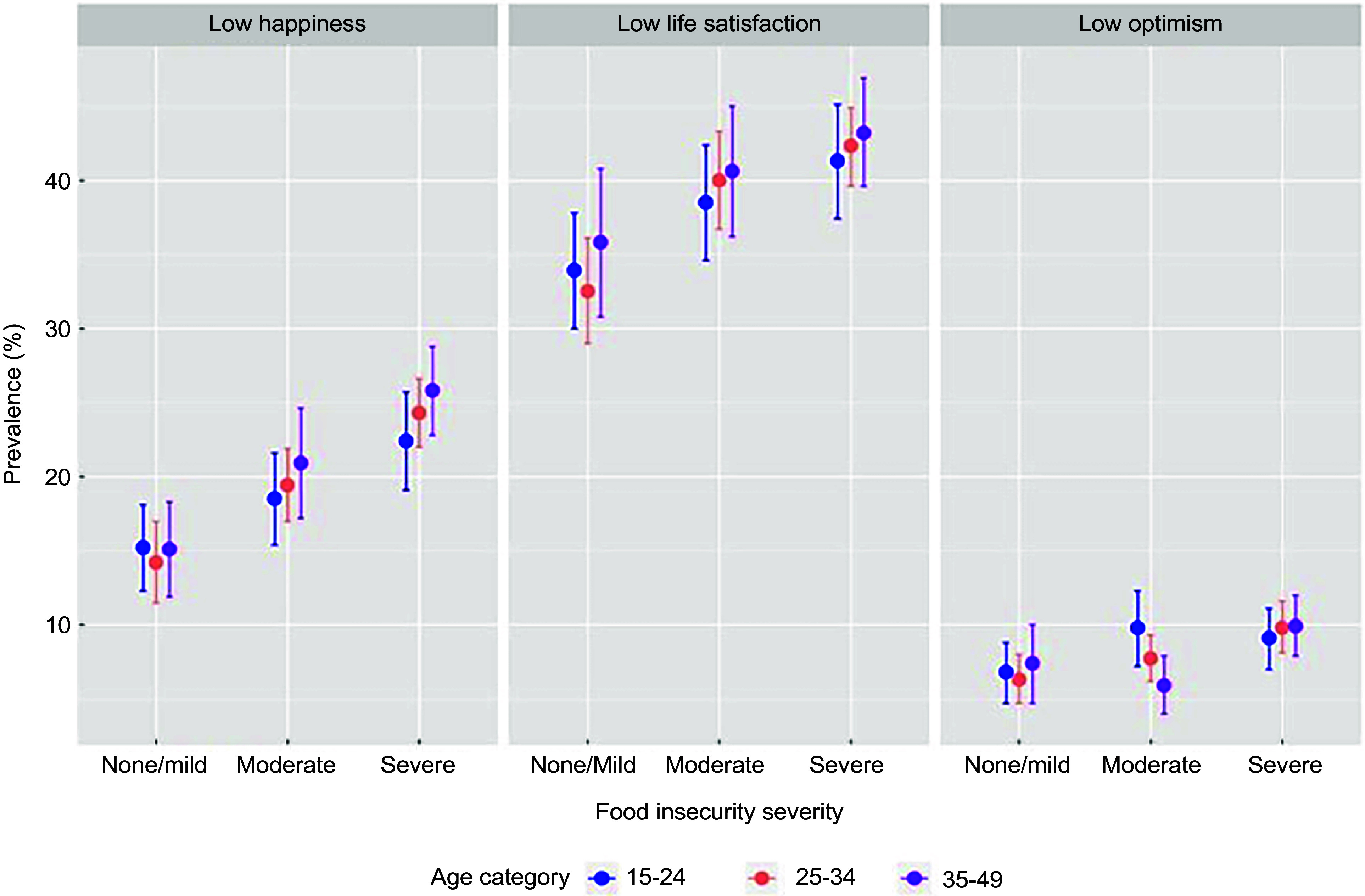
Prevalence of low levels of subjective well-being indicators among women by food insecurity severity, stratified by maternal age. Error bars show 95 % CI.

**Figure 3.**
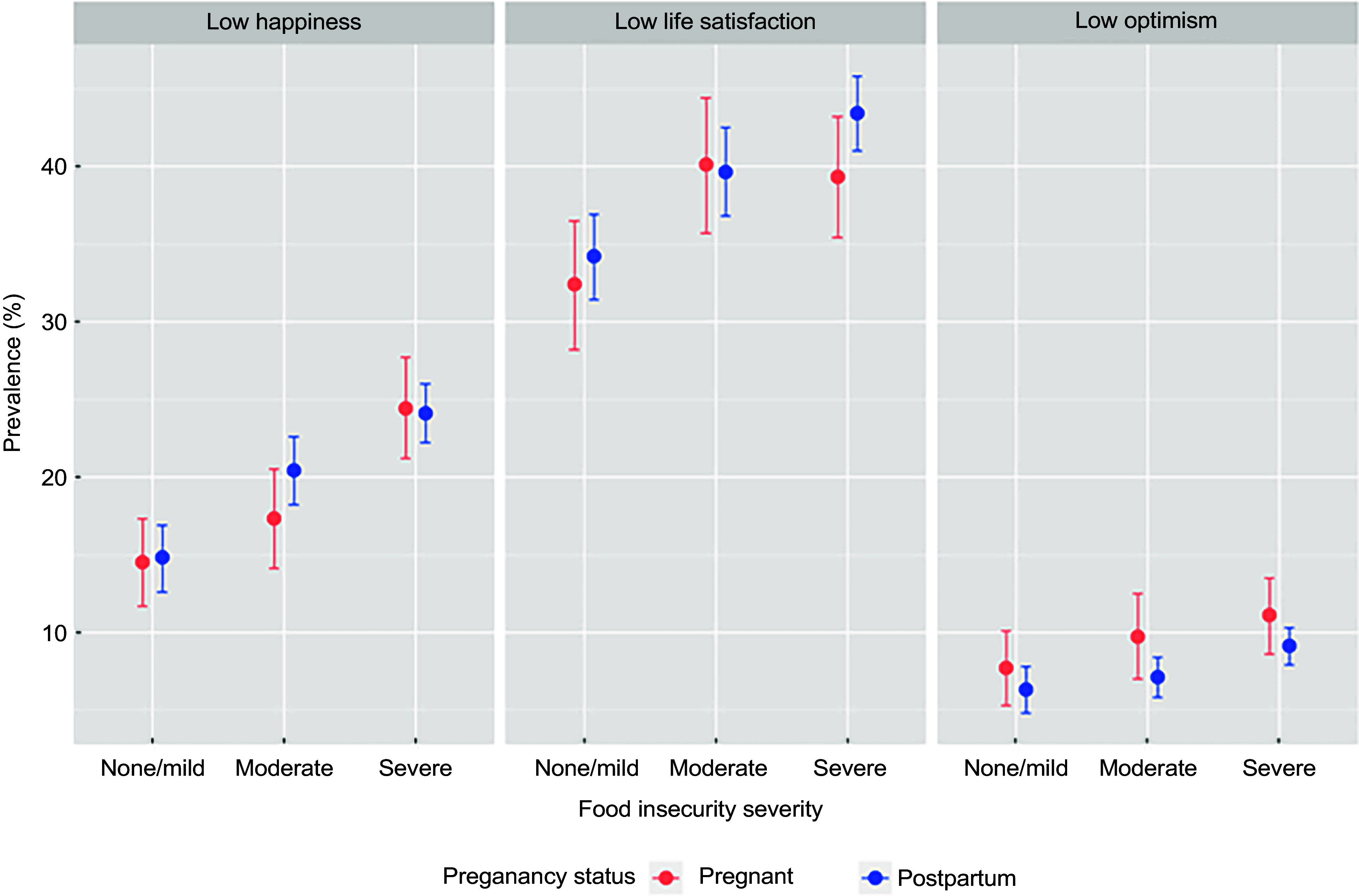
Prevalence of low levels of subjective well-being indicators among women by food insecurity severity, stratified by pregnancy status. Error bars show 95 % CI.

Nearly three-quarters (74·4 %) of respondents were living in households that experienced FI during the past year, comprising 30·5 % moderately and 43·9 % severely food-insecure households (data not shown). As shown in Figure 1, the likelihood of reporting low happiness levels was higher with increasing FI severity (food-secure: 14·7 %, moderate FI: 19·6 % severe FI = 24·7 %; *P* < 0·0001). The prevalence of low life satisfaction was 39·8 % and 42·3 % among those who reported moderate and severe FI, compared with 33·7 % among those who were food-secure (*P* < 0·0001). Compared to their food-secure counterparts, women were also more likely to report low optimism if they were residing in moderately or severely food-insecure households (6·7 % *v*. 7·8 % *v*. 9·6 %; *P* = 0·0003). The prevalence of self-reported low SWB measures and the distribution of food security severity by pregnancy status and by maternal age are presented in Figures 2 and 3, respectively.

### Food insecurity and subjective well-being

At an average community (random effect equal to zero on the logit scale), nearly one-third (27·2 %) of the variability in the likelihood of reporting low happiness levels can be attributed to systematic differences between communities (τ_00_ = 1·2; z(1726) = 11·8, *P* < 0·0001) (Table 2). In model I (Table 2), FI was significantly associated with self-reported low happiness levels. Compared to food-secure women, women experiencing moderate (OR = 1·4, 95 % CI 1·2, 1·6, *P* < 0·0001) and severe FI (OR = 1·7, 95 % CI 1·5, 1·9, *P* < 0·0001) had significantly higher odds of reporting low happiness levels. Based on the model fit statistics, the full model (Table 2, model IV) was the best-fitting model. While there was modest attenuation in the effect estimates, moderate FI was associated with higher odds of reporting low levels of happiness, after adjusting for potential confounders (adjusted OR (aOR) = 1·3, 95 % CI 1·1, 1·4, *P* = 0·0217)

**Table 2.** Weighted multilevel logistic regression models examining the association of food insecurity with low happiness. Multiple Indicator Cluster Survey, Nigeria 2021, (*n* 12 718)

Variables			Model I^†^		Model II^‡^		Model III^§^		Model IV^||^	
	Null model*		OR	95 % CI	aOR	95 % CI	aOR	95 % CI	aOR	95 % CI
Intercept (se)	−1·6***	0·04	−1·9***	0·07	−0·5**	0·16	−2·2***	0·11	−0·5*	0·19
Individual and household characteristics										
Household food insecurity										
None/mild			1·0		1·0		1·0		1·0	
Moderate			1·4	1·2, 1·6	1·3	1·1, 1·5	1·4	1·2, 1·6	1·3	1·1, 1·5
Severe			1·7	1·5, 1·9	1·5	1·3, 1·8	1·7	1·5, 2·0	1·5	1·3, 1·7
Age group, years (range: 15–49)										
15–24					1·0				1·0	
25–34					1·3	1·1, 1·4			1·3	1·1, 1·4
35–49					1·4	1·2, 1·6			1·4	1·2, 1·6
Pregnancy status										
Postpartum					1·0				1·0	
Pregnant					0·9	0·8, 1·0			0·9	0·8, 1·0
Marital status										
Single					1·0				1·0	
Married/living with partner					0·2	0·2, 0·3			0·2	0·2, 0·3
Religion										
Non-Christian					1·0				1·0	
Christian					0·9	0·8, 1·0			0·9	0·7, 1·0
Number of household members										
< 5					1·0				1·0	
≥ 5					0·9	0·8, 1·1			1·0	0·8, 1·1
Household wealth index										
Quintile 1 (lowest)					1·0				1·0	
Quintile 2					0·9	0·8, 1·1			0·9	0·8, 1·1
Quintile 3					0·9	0·8, 1·2			0·9	0·8, 1·1
Quintile 4					0·8	0·6, 0·9			0·7	0·6, 0·9
Quintile 5 (highest)					0·4	0·3, 0·5			0·4	0·3, 0·5
Contextual factors										
Residence										
Rural							1·0		1·0	
Urban							1·2	1·0, 1·4	0·9	0·7, 1·1
Region										
North Central							1·0		1·0	
North East							0·8	0·7, 1·1	0·8	0·6, 1·0
North West							1·4	1·1, 1·7	1·3	1·0, 1·7
South East							1·3	1·0, 1·7	1·3	1·0, 1·8
South South							1·2	0·9, 1·5	1·2	0·9, 1·5
South West							0·9	0·8, 1·3	1·0	0·8, 1·3
Random effects (variance components)										
Cluster-level variance (se)	1·2	0·1	1·2	0·1	1·3	0·1	1·2	0·1	1·2	0·1
ICC (%)	27·2		27·1		27·1		26·4		27·1	
MOR	2·9		2·9		2·9		2·8		2·9	
Model fit statistics										
AIC	12 296·4		12 234·6		11 994·3		12 218·3		11 982·0	
BIC	12 307·3		12 256·4		12 070·6		12 272·8		12 091·1	

aOR, adjusted OR; ICC, intraclass correlation coefficient; MOR, median OR; AIC, Akaike’s information criterion; BIC, Bayesian information criteria.

Random intercepts two-level logistic regression models. Reference category = high happiness levels.

Models were estimated using pseudo-maximum likelihood. Model fit improvement was examined using AIC and BIC. All estimates are weighted for the survey’s complex sampling design.

* *P* < 0.05; ** *P* < 0.01; *** *P* < 0.001.

† Model I: Multilevel model with food insecurity only.

‡ Model II: Multilevel model with food insecurity adjusted for individual-/household-level factors.

§ Model III: Multilevel model with food insecurity adjusted for community-level factors.

|| Model IV: Multilevel model with food insecurity adjusted for individual-/household-level and community-level factors.

As shown in Table 3, the estimate of the total variability in low life satisfaction that occurs between communities was 0·87, resulting in an ICC of 0·21. This suggests that on average, one-fifth of the variability in the likelihood of reporting low life satisfaction is accounted for by the communities in our study (τ_00_ = 0·9; z(1726) = 13·2, *P* < 0·0001). The unadjusted odds of reporting low life satisfaction were 19 % (95 % CI 1·1, 1·4, *P* = 0·0074) and 45 % (95 % CI 1·3, 1·6, *P* < 0·0001) for women experiencing moderate and severe FI, respectively, compared with food-secure respondents (model I, Table 3). However, in the best-fitting model (model IV), only severe FI remained statistically significant after adjustment for confounders (aOR = 1·3, 95 % CI 1·2, 1·5, *P* =< 0·0001).

**Table 3.** Weighted multilevel logistic regression models examining the association of food insecurity with low life satisfaction. Multiple Indicator Cluster Survey, Nigeria 2021, (*n* 12 718)

Variables			Model I^†^		Model II^‡^		Model III^§^		Model IV^||^	
	Null model*		OR	95 % CI	aOR	95 % CI	aOR	95 % CI	aOR	95 % CI
Intercept (se)	0·5***	0·03	−0·7***	0·05	0·4**	0·1	−0·9***	0·1	0·4*	0·2
Individual and household characteristics										
Household food insecurity										
None/mild			1·0		1·0		1·0		1·0	
Moderate			1·2	1·1, 1·4	1·1	1·0, 1·3	1·2	1·1, 1·4	1·1	0·9, 1·3
Severe			1·5	1·3, 1·6	1·3	1·2, 1·5	1·5	1·3, 1·6	1·3	1·2, 1·5
Age group, years (range: 15–49)										
15–24					1·0				1·0	
25–34					1·1	1·0, 1·3			1·2	1·0, 1·3
35–49					1·2	1·1, 1·4			1·2	1·1, 1·4
Pregnancy status										
Postpartum					1·0				1·0	
Pregnant					1·0	0·9, 1·1			1·2	1·1, 1·4
Marital status										
Single					1·0				1·0	
Married/living with partner					0·3	0·3, 0·4			0·3	0·3, 0·4
Religion										
Non-Christian					1·0				1·0	
Christian					0·9	0·8, 1·0			1·0	0·8, 1·1
Number of household members										
< 5					1·0				1·0	
≥ 5					1·0	0·9, 1·2			1·0	0·9, 1·1
Household wealth index										
Quintile 1 (lowest)					1·0				1·0	
Quintile 2					1·0	0·9, 1·1			1·0	0·9, 1·1
Quintile 3					0·9	0·8, 1·0			0·9	0·8, 1·0
Quintile 4					0·8	0·7, 0·9			0·8	0·6, 0·9
Quintile 5 (highest)					0·5	0·4, 0·6			0·5	0·4, 0·6
Contextual factors										
Residence										
Rural							1·0		1·0	
Urban							1·0	0·9, 1·1	0·8	0·7, 0·9
Region										
North Central							1·0		1·0	
North East							1·6	1·3, 1·9	1·5	1·2, 1·8
North West							1·3	1·1, 1·6	1·3	1·1, 1·6
South East							1·3	1·0, 1·6	1·3	1·0, 1·7
South South							1·0	0·8, 1·3	1·0	0·8, 1·2
South West							0·9	0·7, 1·1	0·9	0·7, 1·1
Random effects (variance components)										
Cluster-level variance (se)	0·9	0·1	0·9	0·1	0·9	0·1	0·8	0·1	0·9	0·1
ICC (%)	21·0		20·9		21·3		20·3		20·7	
MOR	2·4		2·4		2·5		2·4		2·4	
Model fit statistics										
AIC	16 328·9		16 283·3		16 117·6		16 255·7		16 097·9	
BIC	16 339·8		16 305·1		16 193·9		16 310·2		16 207·0	

aOR, adjusted OR; ICC, intraclass correlation coefficient; MOR, median OR; AIC, Akaike’s information criterion; BIC, Bayesian information criteria.

Random intercepts two-level logistic regression models. Reference category = high life satisfaction.

Models were estimated using pseudo-maximum likelihood. Model fit improvement was examined using AIC and BIC. All estimates are weighted for the survey’s complex sampling design.

* *P* < 0.05; ** *P* < 0.01; *** *P* < 0.001.

† Model I: Multilevel model with food insecurity only.

‡ Model II: Multilevel model with food insecurity adjusted for individual-/household-level factors.

§ Model III: Multilevel model with food insecurity adjusted for community-level factors.

|| Model IV: Multilevel model with food insecurity adjusted for individual-/household-level and community-level factors.

There was also significant variability across communities in the likelihood of reporting low optimism (τ_00_ = 1·8; z(1726) = 10·9, *P* < 0·0001) (Table 4). At an average community, approximately 35 % of the variability in the likelihood of reporting low optimism is accounted for by the communities in our study. As shown in the crude model, compared with food-secure respondents, the odds of reporting low optimism were 20 % (95 % CI 0·9, 1·5, *P* = 0·097) and 69 % (95 % CI 1·4, 2·1, *P* < 0·0001) higher for women who were moderately and severely food-insecure, respectively. After adjusting for confounders in model IV (best-fitting model), severe FI but not moderate FI was significantly associated with low optimism (aOR = 1·6, 95 % CI 1·3, 2·0, *P* < 0·0001). The full multilevel logistic results for all SWB measures are presented in Tables 2–4.

**Table 4.** Weighted multilevel logistic regression models examining the association of food insecurity with low optimism. Multiple Indicator Cluster Survey, Nigeria 2021, (*n* 12 718)

Variables			Model I^†^		Model II^‡^		Model III^§^		Model IV^||^	
	Null model*		OR	95 % CI	aOR	95 % CI	aOR	95 % CI	aOR	95 % CI
Intercept (se)	−2·9***	0·07	−3·3***	0·11	−2·5***	0·25	−3·6***	0·27	−2·7***	0·3
Individual and household characteristics										
Household food insecurity										
None/mild			1·0		1·0		1·0		1·0	
Moderate			1·2	0·9, 1·5	1·1	0·9, 1·4	1·2	0·9, 1·6	1·2	0·9, 1·5
Severe			1·7	1·4, 2·1	1·6	1·3, 2·0	1·7	1·4, 2·1	1·6	1·3, 2·0
Age group, years (range: 15–49)										
15–24					1·0				1·0	
25–34					1·1	0·9, 1·3			1·1	0·9, 1·3
35–49					0·9	0·8, 1·2			1·0	0·8, 1·3
Pregnancy status										
Postpartum					1·0				1·0	
Pregnant					1·1	1·0, 1·3			1·1	1·0, 1·3
Marital status										
Single					1·0				1·0	
Married/living with partner					0·6	0·4, 0·8			0·5	0·4, 0·7
Religion										
Non-Christian					1·0				1·0	
Christian					0·7	0·6, 0·8			0·8	0·6, 1·0
Number of household members										
< 5					1·0				1·0	
≥ 5					1·2	1·0, 1·4			1·1	0·9, 1·3
Household wealth index										
Quintile 1 (lowest)					1·0				1·0	
Quintile 2					0·8	0·7, 1·1			0·9	0·7, 1·1
Quintile 3					0·8	0·6, 1·0			0·8	0·6, 1·1
Quintile 4					0·7	0·5, 1·0			0·8	0·6, 1·1
Quintile 5 (highest)					0·5	0·3, 0·7			0·6	0·4, 0·9
Contextual factors										
Residence										
Rural							1·0		1·0	
Urban							1·1	0·9, 1·4	1·0	0·7, 1·3
Region										
North Central							1·0		1·0	
North East							2·9	2·2, 4·0	2·6	1·9, 3·6
North West							1·4	1·0, 1·9	1·2	0·9, 1·7
South East							2·3	1·7, 3·3	2·6	1·8, 3·8
South South							0·3	0·2, 0·4	0·3	0·2, 0·5
South West							0·8	0·5, 1·3	0·9	0·6, 1·3
Random effects (variance components)										
Cluster-level variance (se)	1·8	0·2	1·7	0·2	1·7	0·2	1·4	0·1	1·4	0·1
ICC (%)	34·9		34·6		33·6		30·5		29·9	
MOR	3·6		3·5		3·4		3·1		3·1	
Model fit statistics										
AIC	16 328·9		7452·7		7414·8		7302·8		7290·4	
BIC	16 339·8		7474·5		7491·2		7357·3		7399·4	

aOR, adjusted OR; ICC, intraclass correlation coefficient; MOR, median OR; AIC, Akaike’s information criterion; BIC, Bayesian information criteria.

Random intercepts two-level logistic regression models. Reference category = high optimism.

Models were estimated using pseudo-maximum likelihood. Model fit improvement was examined using AIC and BIC. All estimates are weighted for the survey’s complex sampling design.

* *P* < 0.05; ** *p* < 0.01; *** *P* < 0.001.

† Model I: Multilevel model with food insecurity only.

‡ Model II: Multilevel model with food insecurity adjusted for individual-/household-level factors.

§ Model III: Multilevel model with food insecurity adjusted for community-level factors.

|| Model IV: Multilevel model with food insecurity adjusted for individual-/household-level and community-level factors.

### Stratified analyses

#### Food insecurity and subjective well-being by maternal age group

##### Happiness

The stratified analyses show the variations in the prevalence of reported low levels of happiness by FI status across different age categories (Figure 2). The results from the best-fitting model (Table 5, model IV) showed that across all maternal age groups, women experiencing moderate FI were not more or less likely to report low happiness levels compared with their food-secure counterparts. Compared with food-secure women, those experiencing severe FI had approximately 50 % higher odds of reporting low happiness among women aged 15–24 years (aOR = 1·5, 95 % CI = 1·1, 2·0), 25–34 years (aOR = 1·5, 95 % CI = 1·1, 1·9) and 35–49 years (aOR = 1·7, 95 % CI = 1·3, 2·2).

**Table 5.** Multilevel logistic regression models examining the association of food insecurity with measures of SWB by maternal age. Multiple Indicator Cluster Survey, Nigeria 2021, (*n* 12 718)

	Variable	Age, years	Model I^*^		Model II^†^		Model III^‡^		Model IV^§^	
			OR	95 % CI	aOR	95 % CI	aOR	95 % CI	aOR	95 % CI
Happiness	Food insecurity category									
(Reference = high)	Moderate FI (reference = none/mild FI)	15–24	1·4	1·0, 1·8	1·2	0·9, 1·6	1·3	1·0, 1·7	1·2	0·9, 1·6
		25–34	1·4	1·0, 1·8	1·2	0·9, 1·6	1·3	1·0, 1·7	1·2	0·9, 1·6
		35–49	1·4	1·0, 1·9	1·4	0·9, 1·8	1·4	1·0, 1·9	1·3	0·9, 1·8
	Severe FI (reference = none/mild FI)									
		15–24	1·7	1·3, 2·2	1·5	1·1, 2·0	1·6	1·2, 2·2	1·5	1·1, 2·0
		25–34	1·7	1·3, 2·2	1·5	1·1, 1·9	1·6	1·2, 2·2	1·5	1·1, 1·9
		35–49	1·8	1·4, 2·3	1·7	1·3, 2·2	1·8	1·4, 2·3	1·7	1·3, 2·2
Life satisfaction	Food insecurity category									
(Reference = high)	Moderate FI (reference = none/mild FI)	15–24	1·1	0·9, 1·3	1·0	0·8, 1·3	1·1	0·9, 1·4	1·0	0·8, 1·3
		25–34	1·1	0·9, 1·3	1·0	0·8, 1·3	1·1	0·9, 1·4	1·0	0·8, 1·3
		35–49	1·1	0·9, 1·4	1·1	0·8, 1·4	1·1	0·9, 1·5	1·1	0·9, 1·4
	Severe FI (reference = none/mild FI)									
		15–24	1·4	1·2, 1·7	1·3	1·0, 1·6	1·4	1·6, 1·7	1·3	1·1, 1·6
		25–34	1·4	1·2, 1·7	1·3	1·0, 1·6	1·4	1·2, 1·7	1·3	1·1, 1·6
		35–49	1·4	1·1, 1·8	1·4	1·1, 1·8	1·4	1·1, 1·8	1·4	1·1, 1·7
Optimism	Food insecurity category									
(Reference = high)	Moderate FI (reference = none/mild FI)	15–24	1·3	0·9, 1·9	1·3	0·8, 1·9	1·5	0·9, 2·2	1·4	0·9, 2·1
		25–34	1·3	0·9, 1·9	1·3	0·9, 1·9	1·5	0·9, 2·2	1·4	0·9, 2·1
		35–49	1·0	0·6, 1·6	0·9	0·6, 1·6	0·9	0·6, 1·6	0·9	0·6, 1·6
	Severe FI (reference = none/mild FI)									
		15–24	1·5	1·0, 2·2	1·5	1·0, 2·2	1·7	1·2, 2·4	1·5	1·1, 2·3
		25–34	1·6	1·1, 2·2	1·5	1·0, 2·2	1·7	1·2, 2·4	1·5	1·1, 2·3
		35–49	2·0	1·3, 3·2	1·9	1·3, 3·1	1·9	1·2, 3·0	1·9	1·2, 3·0

SWB, subjective well-being; AOR, adjusted OR; FI, food insecurity.

Random intercepts two-level logistic regression models. Due to space limitations, all fixed effects (e.g. age, household wealth index, place of residence, etc.) and random effects were not added to model I–IV notations. Models were estimated using pseudo-maximum likelihood. Model fit improvement was examined using Akaike’s information criterion (AIC) and Bayesian Information Criteria (BIC). All estimates are weighted for the survey’s complex sampling design.

* Model I: Multilevel model with food insecurity only.

† Model II: Multilevel model with food insecurity adjusted for individual-/household-level factors.

‡ Model III: Multilevel model with food insecurity adjusted for community-level factors.

§ Model IV: Multilevel model with food insecurity adjusted for individual-/household-level and community-level factors.

##### Life satisfaction

The stratified analyses reveal the variations in the prevalence of reported low life satisfaction by FI status across different age categories (Figure 2). The results from the best-fitting model (Table 5, model IV) showed that across all maternal age groups, women experiencing moderate FI were not more or less likely to report low life satisfaction compared with their food-secure counterparts. However, compared with food-secure women, those experiencing severe FI had at least 30 % higher odds of reporting low life satisfaction among women aged 15–24 years (aOR = 1·3, 95 % CI = 1·1, 1·6), 25–34 years (aOR = 1·3, 95 % CI = 1·1, 1·6) and 35–49 years (aOR = 1·4, 95 % CI = 1·1, 1·7).

##### Optimism

The stratified analyses reveal the variations in the prevalence of reported low levels of optimism by FI status across different age categories (Figure 2). The results from the best-fitting model (Table 5, model IV) showed that across all maternal age groups, women experiencing moderate FI were not more or less likely to report low levels of optimism compared with their food-secure counterparts. However, compared with food-secure women, those experiencing severe FI had at least 50 % higher odds of reporting low levels of optimism among women aged 15–24 years (aOR = 1·5, 95 % CI = 1·1, 2·3), 25–34 years (aOR = 1·5, 95 % CI = 1·1, 2·3) and 35–49 years (aOR = 1·9, 95 % CI = 1·2, 3·0).

#### Food insecurity and subjective well-being by pregnancy status

##### Happiness

The stratified analyses reveal the variations in the prevalence of reported low levels of happiness by FI status and pregnancy status (Figure 3). Based on the best-fitting model (Table 6, model IV), the association between FI and reported low levels of happiness varies based on pregnancy status. In the Level 1 and Level 2 confounder-adjusted model (model IV), we observed that, for postpartum women, there was a significant association between moderate FI and reported low levels of happiness (aOR = 1·3, 95 % CI = 1·1, 1·6). However, this association was not significant for pregnant women.

**Table 6.** Multilevel logistic regression models examining the association of food insecurity with measures of SWB by pregnancy status. Multiple Indicator Cluster Survey, Nigeria 2021 (*n* 12 718)

	Variable	Status	Model I^*^		Model II^†^		Model III^‡^		Model IV^§^	
			OR	95 % CI	aOR	95 % CI	aOR	95 % CI	aOR	95 % CI
Happiness	Food insecurity category									
(Reference = high)	Moderate FI (reference = none/mild FI)	Pregnant	1·4	1·0, 1·8	1·3	0·9, 1·7	1·3	0·9, 1·8	1·2	0·9, 1·7
		Postpartum	1·5	1·2, 1·7	1·3	1·1, 1·6	1·4	1·2, 1·7	1·3	1·1, 1·6
	Severe FI (reference = none/mild FI)	Pregnant	1·7	1·3, 2·2	1·5	1·1, 1·9	1·7	1·3, 2·2	1·5	1·1, 2·0
		Postpartum	1·8	1·5, 2·1	1·5	1·3, 1·8	1·7	1·5, 2·1	1·5	1·3, 1·8
Life satisfaction	Food insecurity category									
(Reference = high)	Moderate FI (reference = none/mild FI)	Pregnant	1·4	1·1, 1·8	1·4	1·1, 1·7	1·4	1·1, 1·8	1·4	1·1, 1·7
		Postpartum	1·1	0·9, 1·3	1·1	0·9, 1·2	1·1	0·9, 1·3	1·1	0·9, 1·2
	Severe FI (reference = none/mild FI)	Pregnant	1·3	1·1, 1·6	1·2	0·9, 1·5	1·3	1·1, 1·6	1·2	0·9, 1·5
		Postpartum	1·5	1·3, 1·8	1·4	1·2, 1·6	1·5	1·3, 1·8	1·4	1·2, 1·6
Optimism	Food insecurity category									
(Reference = high)	Moderate FI (reference = none/mild FI)	Pregnant	1·2	0·8, 1·9	1·2	0·8, 1·8	1·2	0·9, 1·9	1·2	0·8, 1·9
		Postpartum	1·2	0·9, 1·5	1·1	0·8, 1·4	1·2	0·9, 1·5	1·1	0·9, 1·5
	Severe FI (reference = none/mild FI)	Pregnant	1·6	1·1, 2·2	1·5	1·0, 2·2	1·6	1·1, 2·3	1·5	1·0, 2·2
		Postpartum	1·8	1·4, 2·3	1·6	1·3, 2·1	1·8	1·4, 2·3	1·6	1·3, 2·1

SWB, subjective well-being; AOR, adjusted OR; FI, food insecurity.

Random intercepts two-level logistic regression models. Due to space limitations, all fixed effects (e.g. age, household wealth index, place of residence, etc.) and random effects were not added to model I–IV notations. Models were estimated using pseudo-maximum likelihood. Model fit improvement was examined using Akaike’s information criterion (AIC) and Bayesian Information Criteria (BIC). All estimates are weighted for the survey’s complex sampling design.

* Model I: Multilevel model with food insecurity only.

† Model II: Multilevel model with food insecurity adjusted for individual-/household-level factors.

‡ Model III: Multilevel model with food insecurity adjusted for community-level factors.

§ Model IV: Multilevel model with food insecurity adjusted for individual-/household-level and community-level.

Both pregnant and postpartum women experiencing severe FI, compared to their counterparts experiencing no FI, had significantly higher odds of reporting low levels of happiness. Specifically, severe FI was associated with 50 % higher odds of reporting low levels of happiness among pregnant women (aOR = 1·5, 95 % CI = 1·1, 2·0) and postpartum women (aOR = 1·5, 95 % CI = 1·3, 1·8), after adjusting for confounders.

##### Life satisfaction

The stratified analyses show the variations in the prevalence of reported low levels of life satisfaction by FI status and pregnancy status (Figure 3). Based on the best-fitting model (Table 6, model IV), the association between FI and reported low life satisfaction varies based on pregnancy status. In the Level 1 and Level 2 confounder-adjusted model (model IV), we observed that, for pregnant women, there was a significant association between moderate FI and reported low levels of low life satisfaction (aOR = 1·4, 95 % CI = 1·1, 1·7). However, this association was not significant for postpartum women. On the other hand, pregnant women experiencing severe FI, when compared to those experiencing no FI, had 40 % higher odds of reporting low levels of low life satisfaction (aOR = 1·4, 95 % CI = 1·2, 1·6).

##### Optimism

The stratified analyses demonstrate the variations in the prevalence of reported low levels of optimism by FI status and pregnancy status (Figure 3). Based on the best-fitting model (Table 6, model IV), the association between FI and reported low optimism levels varies based on pregnancy status. In the Level 1 and Level 2 confounder-adjusted model (model IV), we observed that moderate FI was not associated with the odds of reporting low optimism levels, regardless of pregnancy status. On the other hand, pregnant women experiencing severe FI, when compared to those experiencing no FI, had 40 % higher odds of reporting low optimism levels (aOR = 1·5, 95 % CI = 1·0, 2·2), while postpartum women experiencing severe FI, when compared to those experiencing no FI, had a 60 % higher odds of reporting low optimism levels (aOR = 1·6, 95 % CI = 1·3, 2·1).

## Discussion

We observed that approximately three out of every four peripartum women were experiencing FI. Consistent with our hypothesis, we observed that peripartum women who were exposed to any level of FI were more likely to report low levels of happiness, life satisfaction and optimism, after adjusting for individual and structural factors compared to their counterparts who were food-secure. Moreover, the likelihood of reporting low levels of measures of SWB tended to be higher with increasing severity of FI which is consistent with the findings by Asfahani *et al.* ^(30)^ who also showed the correlation between increasing levels of FI and higher well-being inequality.

Although moderate FI was initially associated with low life satisfaction in the crude model in our study, we did not find a statistically significant difference for this association in the best-fitting model adjusting for confounders. In the stratified analyses, we observed significant heterogeneity in the association between FI and low levels of SWB measures based on maternal age and pregnancy status. This suggests that maternal age and pregnancy status modify the relationship between FI and SWB. While the associations varied based on different maternal age and pregnancy categories, they remained consistent for all age groups and pregnancy statuses for women experiencing severe FI.

Regarding life satisfaction, several studies have demonstrated a significant and negative correlation between FI and overall life satisfaction. For instance, Sulemana and James^(39)^ revealed that in five sub-Saharan African countries, including Nigeria, individuals with higher levels of FI were less likely to report satisfaction with their lives. Similarly, a study by Selvamani *et al.* showed that older adults facing severe FI were 2·36 times more likely to report lower levels of life satisfaction^(19)^. Additionally, within Aboriginal populations in Canada, Willows *et al.* ^(40)^ found a higher likelihood of reporting low life satisfaction among individuals experiencing FI. Although there are limited studies examining the association between FI and SWB specifically in pregnant and postpartum women, one study conducted among socio-economically disadvantaged individuals across three urban areas of Indonesia found that those experiencing FI were more likely to report lower levels of happiness^(41)^.

In our study, we observed that the association of severe FI with happiness exhibited greater magnitude and strength compared to the other measures of SWB. However, this finding contrasts with the results presented in a study by Frongillo *et al.* ^(42)^, where the magnitude of the association of FI was stronger for life satisfaction.

In our age-stratified analyses, we consistently observed a higher likelihood of reporting low levels of SWB across all domains (excluding optimism) among those experiencing severe FI across all age groups. For women facing moderate FI, the likelihood of reporting low levels of SWB across all three measures was significantly higher only for pregnant and postpartum women aged 25–34 years. These findings are consistent with evidence from a previous study involving youth across various countries in the Arab region which revealed that both moderate and severe FI correlated with lower life evaluation scores^(30)^.

Furthermore, our analysis indicates that although the relationships of moderate FI on happiness and optimism appeared somewhat similar among pregnant and postpartum women, we did not find any substantial evidence of a significant association with life satisfaction for either group. These findings might suggest that life satisfaction during pregnancy and postpartum was less likely to be impacted by moderate FI. This underscores the importance of considering individual context and unique circumstances when designing and implementing evidence-based strategies to address FI among pregnant and postpartum women of reproductive age.

While the findings from our study are largely consistent with the hypothesised relationship between FI and poor well-being^(43)^, they raise important concerns regarding the potential implications for expectant and new mothers and their children. Our results align with a growing body of evidence demonstrating strong associations between FI and adverse maternal mental health outcomes. For example, a recent scoping review synthesising evidence across multiple settings found that prenatal FI is consistently associated with increased risks of depression, anxiety and stress during pregnancy and the postpartum period, with all included studies reporting positive associations between FI and adverse perinatal mental health outcomes^(44)^. Similarly, analyses of large population-based datasets have reported more than threefold higher odds of antepartum depressive symptoms among food-insecure women, as well as graded increases in anxiety severity associated with FI^(35,45)^.

Beyond its effects on maternal well-being, HFI has also been linked to adverse child health and developmental outcomes. Evidence suggests that children living in food-insecure households face increased risks of developmental delays and poorer health indicators. For instance, a study among mother–infant pairs in Brazil found that infants living in food-insecure households had significantly higher odds of experiencing early developmental delays compared with those in food-secure households^(40)^. Notably, these associations were substantially stronger among children whose mothers experienced depression or anxiety, indicating that maternal psychological distress may amplify the adverse effects of FI on child outcomes^(46)^. These findings underscore the importance of addressing FI among pregnant and postpartum women in Nigeria. Given growing evidence that FI, regardless of severity, is associated with multiple dimensions of SWB, multilevel and multidimensional strategies aimed at reducing FI among pregnant and postpartum women could substantially improve maternal well-being and, ultimately, maternal, fetal and child health outcomes.

### Research, policy and practice implications

The findings from this study have implications for public health. Future research efforts should focus on elucidating the mechanisms through which diminished levels of SWB among women experiencing FI influence outcomes related to pregnancy and the postpartum period. While we aimed to approximate a life course approach, longitudinal studies will be better suited to investigate the short-and long-term influences of FI on maternal SWB across the life course and critical periods of development as highlighted out by Brown *et al.* ^(13)^. Furthermore, additional research could perhaps adopt a more comprehensive approach to assessing FI by incorporating measures that capture all four dimensions of FI. While this study focused on food access (based on the FIES), future investigations could integrate indicators of food availability (e.g. local production and market supply), food utilisation (e.g. dietary diversity, nutritional adequacy and food safety) and stability (e.g. seasonal or economic fluctuations in food supply and access). Employing multidimensional tools or combining existing measures such as the FIES with dietary recall or market-level data would provide a more nuanced understanding of the mechanisms through which FI influences SWB^(47)^. Relatedly, it would also be worthwhile to leverage qualitative research designs to complement quantitative measures of SWB. Through in-depth interviews, focus groups or ethnographic approaches, qualitative methods can capture the nuanced and lived experiences of women as they navigate different dimensions of FI^(48)^. Such approaches allow for a richer understanding of how the availability, access, utilisation and stability of food, both independently and synergistically, influence various aspects of SWB. By highlighting the contextual and emotional dimensions often missed by standardised tools, qualitative insights could help refine measurement frameworks and guide the development of more holistic interventions.

Given that our study demonstrates the potential relationship of severe FI with well-being of pregnant and postpartum women, it is imperative to design policies aimed at lifting these women out of FI, particularly its most severe forms. Furthermore, policy initiatives centred around establishing food assistance programmes tailored specifically for pregnant and postpartum women play a crucial role in promoting optimal well-being across the trajectory of maternal and child development, as emphasised by Selvamani *et al.* ^(19)^. In cases where such programmes are already in existence, a critical evaluation of their effectiveness is essential to identify potential gaps that warrant attention. Unfortunately, recent systematic reviews focusing on FI screening methods within reproductive healthcare contexts^(49)^ and interventions addressing FI among pregnant women and new mothers^(50)^ indicate an explicit absence of such initiatives for pregnant and postpartum women in Nigeria.

Given the burden of FI among pregnant and postpartum women, there is a need to design innovative and contextually appropriate multilevel interventions and practical strategies, both at health and community settings. Thus, it is important for women’s healthcare providers to incorporate FI screening as an integral part of their assessment process when evaluating women’s well-being^(22)^. Through this, healthcare providers can effectively identify women who may require referrals to community-based programmes and targeted interventions to address FI.

### Strengths and limitations

This study has several strengths. Through the adoption of a social-ecological approach, our research fills an important evidence gap by investigating the influence of contextual factors on the link between FI and well-being. Moreover, as our study used clearly defined and validated tools to capture FI and SWB, we provide compelling evidence on the FI–SWB relationship, facilitating comparability across studies and contexts. Such evidence is important for driving progress towards achieving the SDG. Furthermore, this study comprehensively evaluates SWB across multiple domains, including optimism, which has been rarely investigated in the context of FI. This approach provides valuable insights into which aspects of well-being are susceptible to the effects of FI.

Our study had several limitations. First, the FIES used in our analysis captures FI at the household level and across varying levels of severity. However, as FIES primarily measures food access, our study and the related findings do not account for the potentially distinct effects of the other dimensions of FI on SWB. Second, the MICS lacks additional information about pregnancies, such as gestational age, for pregnant women. This limitation hinders our ability to accurately assess the potential effects of FI on SWB across different pregnancy stages. Third, although self-reported measures are widely used to assess FI and SWB, it is important to acknowledge the inherent bias associated with relying on individuals’ recollections of their experiences.

### Conclusion

Our study shows that HFI was associated with higher odds of reporting low levels of SWB among the maternal population in Nigeria. While ensuring that the health and well-being of pregnant and postpartum women remains a public health priority, efforts aimed at identifying food-insecure women and developing interventions to promote food security in households will help to improve the overall well-being during pregnancy and postpartum.
